# Curcumin Electroanalysis at a Disposable Graphite Electrode

**DOI:** 10.3390/bios15030137

**Published:** 2025-02-23

**Authors:** Mihaela-Carmen Cheregi, Alina Tirsoaga, Cosmina Ion, Emilia-Elena Iorgulescu, Iulia Gabriela David, Hassan Noor

**Affiliations:** 1 Department of Analytical Chemistry and Physical Chemistry, Faculty of Chemistry, University of Bucharest, Panduri Av. 90-92, District 5, 050663 Bucharest, Romania; 2 Department of Surgery, Faculty of Medicine, “Lucian Blaga” University Sibiu, Lucian Blaga Street 25, 550169 Sibiu, Romania; 3 Medlife-Polisano Hospital, Strada Izvorului 1A, 550172 Sibiu, Romania

**Keywords:** curcumin, pencil graphite electrode, voltammetry, dietary supplements

## Abstract

Curcumin (CU, turmeric), a polyphenolic phytochemical that is largely used as a food spice, has benefits for human health, which have led to increased interest in its therapeutic applications and its analysis from different matrices. The two guaiacol moieties of CU are responsible for its antioxidant properties and allow for its voltammetric quantification. Cyclic and differential pulse voltammetry (DPV) investigations at a single-use pencil graphite electrode (PGE) emphasized complex pH-dependent electrode processes, involving an equal number of protons and electrons. Theoretical calculations predicted a folded geometry for the β-diketone CU conformers, which interact with the PGE surface, exposing the electroactive moieties of only one aromatic ring. The Gibbs energy variations of the structures involved in CU electro-oxidation and the theoretical electrochemical potential values were calculated. CU’s DPV cathodic peak intensity recorded at an HB-type PGE in 0.05 mol × L^−1^ H_2_SO_4_ varied linearly in the range 5.00 × 10^−8^–5.00 × 10^−6^ mol × L^−1^ CU. The method’s detection and quantification limits were 2.12 × 10^−8^ mol × L^−1^ and 6.42 × 10^−8^ mol × L^−1^, respectively. The practical applicability of the developed method, successfully tested by CU assessment in dietary supplements, provided a recovery of 99.28 ± 2.04%.

## 1. Introduction

**Figure 1:**
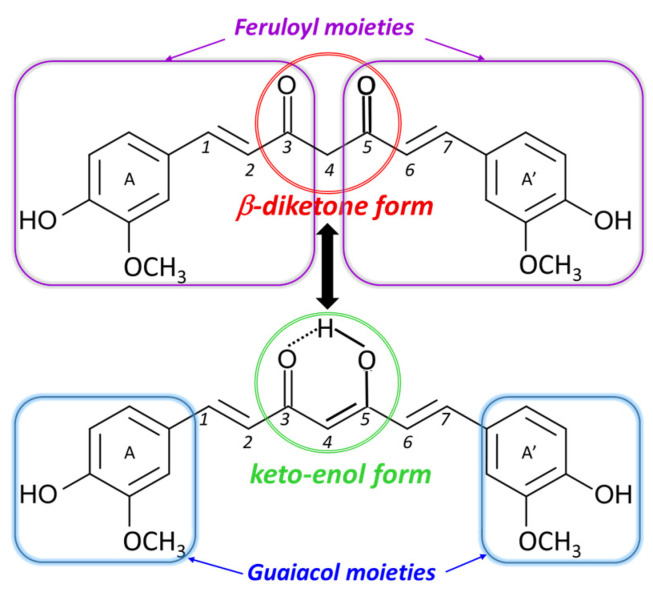
Curcumin’s chemical structure and its tautomeric structures [1].

Curcumin (CU), also known as diferuloylmethane or [(1*E*,6*E*)−1,7−bis(4−hydroxy−3-methoxyphenyl)hepta−1,6−diene−3,5−dione], is a yellow pigment extracted from *Curcuma longa* rhizomes. Depending on the solvent, pH, and temperature, CU can exist in two tautomeric forms (Figure 1), namely, the β-diketone form, composed of two feruloyl groups connected through a methylene group, and the keto-enol form, in which two guaiacol groups are linked by a bridge consisting of seven carbon atoms bearing an α,β-unsaturated β-diketone moiety, all of them contributing to CU’s multiple biological activities [1].

CU is a natural nutraceutical that is well known due to its antioxidant [2,3], anti-inflammatory [4], antibacterial [5], and antitumor properties [6,7,8], being efficient in the prevention and treatment of different types of cancer, e.g., colorectal [9,10]. Recent reports have also discussed CU’s therapeutic effects in neurodegenerative diseases like Alzheimer’s and Parkinson’s diseases [11,12]. Unfortunately, CU’s low solubility reduces its bioavailability and, therefore, its biological effects. At present, there are many investigations directed towards finding solutions to minimize this drawback, and different ways have already been developed to increase CU’s bioavailability and its targeted release [2,3,4,6,7,8].

Since ancient times, CU has been used as a spice or food additive, but today, due its important biological effects, it is commercialized as dietary herbal supplements, or it is applied to cosmetics or intelligent food packaging [5]. However, some studies have indicated that consuming large doses of CU may pose some health risks. Therefore, the daily intake of CU recommended by the WHO is up to 3 mg × kg^−1^ body weight [13]. Consequently, it is important to develop reliable methods for CU quantification in various samples. However, before its analysis from complex matrices, most often, an extraction step is necessary, and the selection of the proper solvent is often crucial [14,15]. The literature reports various analytical methods applied for the analysis of this important polyphenolic compound. The spectral characteristics of CU extracted from *Curcuma longa* were assessed by UV–visible absorption, FT-IR, micro-Raman, and GC–MS methods [16]. A simple UV–visible spectrophotometric method, based on CU’s absorption maxima at 418 nm, was validated and applied to CU assessment in polyherbal formulations [17]. Starting from the reaction of CU with FeCl_3_, which produces a brownish color, a method based on smartphone digital image colorimetry was recently reported for the simple and rapid semi-quantitative estimation of CU in turmeric. This method presented a linear range from 0.25 to 5 mg × L^−1^ and an LOD of 0.12 mg × L^−1^ CU [18].

CU’s ability to quench the fluorescence of MoS_2_ QDs [19], N-MoS_2_ QDs [20], and NCDs [13] has been exploited in the development of fluorimetric methods for its quantification in the concentration ranges 0.10–20 μg × L^−1^ CU, 1.00 × 10^−7^–1.00 × 10^−5^ mol × L^−1^ CU, and 8.10 × 10^−8^–5.15 × 10^−5^ mol × L^−1^ CU, respectively. The attained LODs were 5 ng × L^−1^ CU, 5.9 × 10^−8^ mol × L^−1^ CU, and 1.59 × 10^−8^ mol × L^−1^ CU, respectively. Using a fluorescence imaging 3D-printed smartphone platform, NCDs were also used for the detection of CU concentrations between 1.30 × 10^−7^ and 5.40 × 10^−5^ mol × L^−1^, and with an LOD of 1.32 × 10^−7^ mol × L^−1^ CU.

An RP_HPLC method with UV detection was developed for the analysis of CU in bulk drug form and in nanoemulsion [21], while HPTLC with UV–visible detection was applied for its quantification in a marked polyherbal formulation [22]. RP_HPLC methods have been also applied for the simultaneous quantification of CU and piperine in microparticle formulations containing *C. longa* and *P. nigrum* [23], in human intestinal epithelial cell lysates and mouse plasma, and to investigate CU’s bioavailability in the presence of piperine [24].

All of these techniques are sensitive and selective, but they are reagent- and time-consuming and require expensive instrumentation and trained personnel. A simpler and more cost-effective alternative to these is represented by the electrochemical techniques, which are proper for the examination of chemical species that can be oxidized or reduced at a suitable electrode. CU is a polyphenolic compound that is easily oxidized. Therefore, voltammetry is a good alternative for the simple and rapid detection of this phytochemical. The literature is rich in studies regarding CU’s electrochemistry, and the papers on this topic were recently reviewed by David et al. [1] and Chiorcea-Paquim [25]. Moreover, electrochemical methods are also important tools for the investigation of various interactions between important biological molecules, being helpful in the understanding of some biological processes. Thus, DPV and CV were used to investigate the temperature-dependent interaction between dsDNA and CU, which could bring new insights into the hyperthermia treatment of cancers [26]. To develop a DNA biosensor that could be used in cancer research, CU’s interactions with nucleic acids and PCR samples were monitored electrochemically by measuring the changes in CU and guanine’s oxidation peaks, respectively, at a disposable HaP-IL/PGE [27].

The literature reports several electrodes for CU analysis, most of them being modified with various materials. The present paper describes the electroanalysis of CU and its sensitive quantification at a bare PGE. To the best of our knowledge, this is the first report on using the cheap, disposable, and commonly available PGE as an eco-friendly and versatile tool for the rapid and simple screening of CU in polyherbal dietary supplements

## 2. Materials and Methods

- **Reagents and solutions**

All used reagents were of analytical grade and were purchased from Merck KGAA (Darmstadt, Germany). All aqueous solutions were prepared in double-distilled water.

The stock solution of 1.00 × 10^−3^ mol × L^−1^ CU in ethanol was always freshly prepared on the day of use.

The electrochemical pretreatment of the PGE was performed in ABS pH 3.50 and PBS pH 7.00. For the investigation of the influence of pH and the nature of the supporting electrolyte on CU’s voltammetric response, BRB solutions with pH values in the range 1.81 to 9.15, 0.10 mol × L^−1^ HCl, 0.10 mol × L^−1^ HNO_3_, and 0.05 mol × L^−1^ H_2_SO_4_ were used.

The practical applicability of the developed voltammetric method was tested by determining the CU content of herbal dietary supplement capsules (Curcumin95, produced by AdNatura). The content of these capsules declared by the manufacturer was as follows: curcumin extract from *Curcuma longa* rhizomes with minimum 95% curcuminoids, representing 355 mg and black pepper fruit extract (*Piper nigrium*), with a minimum of 5% piperine.

The contents of three capsules of the herbal dietary supplement Curcumin95 were weighed and finely ground, the mass being denoted as m_caps_. The amount (m_sample_) necessary to produce a 10 mL stock solution with a theoretical concentration of 1.00 × 10^−3^ mol × L^−1^ CU (C_sample_) was weighed from the obtained powder and dissolved in ethanol in a 10 mL volumetric flask. After sonication for 10 min, to ensure complete dissolution of the CU, the solution was filtered. Using a micropipette, an aliquot sample (2.5 μL) was taken from the filtrate to prepare a 10 mL working solution with a theoretical concentration of 2.50 × 10^−7^ mol × L^−1^ CU in 0.05 mol × L^−1^ H_2_SO_4_. To reduce the matrix effects, the method of successive standard additions was applied by adding 3 × 0.10 mL of an intermediate stock solution with a concentration of 1.00 × 10^−5^ mol × L^−1^ CU in ethanol. From the regression equation describing the dependence of the peak currents recorded before and after each addition on the concentration of added CU (I_pc1_ = aC_ad_ +b), the CU concentration in the diluted sample solution (C_dil,sample_) was assessed as follows:

(1) $$C_{dil,sample}=\left|-\frac{b}{a}\right|$$

Considering the dilution performed, the CU concentration in the undiluted sample solution (C_sample_) was calculated as follows:

(2) $$C_{sample}=\frac{10\times C_{dil,sample}}{2.5\times 10^{-3}}\;\;$$

The mass of CU contained in the 10 mL sample solution (m_CU,sample_) was calculated considering CU’s molecular weight (MCU = 366.68 g × mol^−1^), as follows:

m_CU,sample_ = 3.6868 × C_sample_ (3)

Knowing the amount of capsule powder taken for analysis (m_sample_) and the whole mass of the analyzed capsules’ content (m_caps_), the CU content of the analyzed capsules (m_CU,caps_) was calculated:

(4) $$m_{CU,caps}=\frac{m_{caps}}{m_{sample}}\times m_{CU,sample}$$

The % recovery was assessed by comparing the obtained result (m_CU,caps_) with the content declared by the producer, applying the following relation:

(5) $$R\%=\frac{m_{CU,caps}}{3\times 0.355}\times 100$$

- **Instrumentation:**

Voltammetric recordings were performed using a PGSAT 12 potentiostat/galvanostat (Metrohm-Autolab, Utrecht, the Netherlands), using the GPES 4.9. software program, connected to a voltammetric cell consisting of a PGE as the working electrode (if not stated otherwise), a Ag/AgCl/KCl (3.00 mol × L^−1^) reference electrode, and a Pt wire acting as a counter electrode. The electroactive part of the working electrode was represented by a graphite pencil lead (diameter 0.5 mm, Rotring Ticky, China) while a mechanical pencil holder had the role of electrode support. The geometric surface area of the PGE was always 0.1589 cm^2^, while that of the GCE, used for comparison, was 0.0707 cm^2^. The procedure for PGE preparation was as described previously [28].

- **Theoretical modeling:**

Theoretical calculations were performed using Orca software [29,30] version 6. As a general overview, our theoretical study began with a search for the lowest-energy CU conformer using a global optimization algorithm that relies on the external helper program xTB [31], which implements extended tight binding of 2nd-level GFN methods [32] focused on molecular properties such as geometries, frequencies, and noncovalent interactions. The obtained geometries were further optimized by DFT-level calculations [33] using the B3LYP hybrid-type functional (including Grimme’s dispersion correction [34,35,36]) with Karlsruhe/Ahlrichs def2-SVP and, subsequently, -TZVP basis sets [37], while also performing the corresponding Hessian calculations to confirm the reach of a local minimum (no imaginary frequencies), and to assess the thermochemical properties. To mitigate the DFT computational effort, the chain-of-spheres algorithm [38] and the resolution-of-identity [39] approximations [40] were used. Calculations were performed in vacuo or in a condensed medium (water solution) using the conductor-like polarizable continuum model (CPCM) [41]. Higher-level coupled-clusters single-point calculations (CCSD(T) with domain-based local pair natural orbital (DLPNO) scaling methodology [42,43,44,45,46,47]) were also run (using the def2 QZVPP basis set) for selected structures to obtain a more accurate electronic energy value, which was further used to better assess the thermochemical properties subsequently used to calculate the electrochemical potentials. Images were rendered from the Molden [48] or Jmol [49] graphical user interfaces.

## 3. Results and Discussion

The first step in developing an analytical method is the optimization of the measurement conditions to obtain the highest sensitivity and the best selectivity of the determination. For a voltammetric method, the most important chemical parameters affecting the recorded signal are the working electrode and the supporting electrolyte.

### 3.1. Selection of the Optimal Conditions for the Voltammetric Determination of CU

#### 3.1.1. The Working Electrode

- The influence of the electrode material

The study of the voltammetric behavior and the quantitative determination of a chemical species usually begin with the selection of the optimal working electrode. At this stage, the voltammetric signals of the analyte of interest, recorded on different working electrodes, are compared. Thus, the anodic response of CU was investigated on carbon-based working electrodes, namely, the commonly used solid GCE and disposable PGE, using graphite leads with various hardness (2B, B, HB, and H), characterized by different contents of graphite in the composite material constituting the pencil lead [50]. According to the literature, the mass percentages of graphite, clay, and wax in the employed pencil leads are 74:20:5, 71:23:5, 68:26:5, and 63:21:5 for the 2B, B, HB, and H grades, respectively [51]. In the same supporting electrolyte, the anodic response of CU consisted of two unresolved peaks, situated at almost the same potentials, regardless of the working electrode, but with slightly different intensities. The more anodic signal, which was always positioned at around 0.670 V vs. Ag/AgCl/KCl (3.00 mol × L^−1^) (Table 1), with the highest intensity, was further considered for the evaluation of the most suitable working electrode. Because the height of the voltammetric signal depends on both the concentration of the analyte and the area of the electroactive surface of the electrode, the sensitivity, S (A × L × mol^−1^ × cm^−2^), was employed to characterize the voltammetric performance of the electrode material relative to CU. Since the PGE using HB-type graphite leads (denoted as PGE_HB) presented the highest S (Table 1), this was selected as the working electrode for the subsequent studies.

- Electrochemical pretreatment of the electroactive electrode surface:

The electrochemical properties of the working electrode may be improved by modification of its electroactive surface. This can be achieved in multiple ways, most of them being time- and reagent-consuming. The simplest and most eco-friendly manner to change the performance characteristics (sensitivity and selectivity) of the working electrode is its electrochemical pretreatment, or electroactivation, performed by applying high potentials [50]. In the present study, the PGE_HB was activated both potentiodynamically, by CV performed for 10 cycles of potential in the range 0.000–2.000 V vs. Ag/AgCl/KCl (3 mol × L^−1^) with a scan rate of 0.100 V × s^−1^, and potentiostatically, by applying a potential of 2.000 V vs. Ag/AgCl/KCl (3.00 mol × L^−1^) to the working electrode for 60 s. ABS pH 3.50 and PBS pH 7.00 were used as supporting electrolytes in the electrochemical pretreatment process. The electroactivated electrodes were used for the DPV analysis of a solution of known CU concentration. The peak currents obtained on each electroactivated electrode were compared with those recorded at the non-activated PGE. One can see from Figure S1 that, due to electrochemical pretreatment of the electrodes, the pre-wave diverges, and a single, rather wide anodic signal is obtained. Since potentiodynamic activation in ABS pH 3.50 led to the highest oxidation signal of CU, further studies were performed with PGEs pretreated by CV in ABS pH 3.50 (PGE_HB*).

#### 3.1.2. The Supporting Electrolyte

The redox behavior of a chemical species is affected by the pH and the nature of the supporting electrolyte, especially if the analyte contains ionizable functional groups, as is the case with CU, whose dissociation equilibria are pH-dependent.

**Figure 2:**
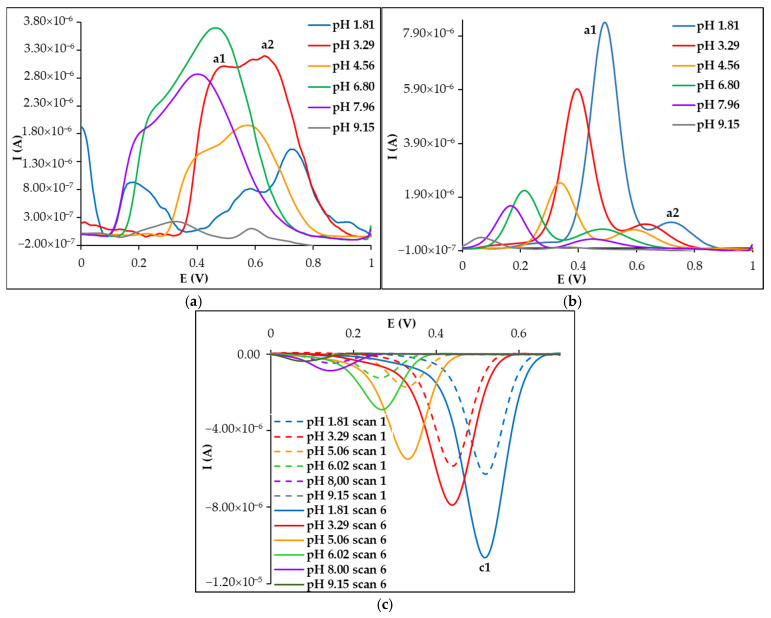
Differential pulse voltammograms recorded at PGE_HB for 5.00 × 10^−6^ mol × L^−1^ CU in BRB solutions with different pH values: (**a**) anodic direction—scan 1; (**b**) anodic direction—scan 6; (**c**) cathodic direction—scan 1 and scan 6.

- The influence of the solution pH:

The influence of the pH of the supporting electrolyte on both the anodic and cathodic voltammetric behavior of CU was investigated in the pH range 1.81 to 9.15, using universal BRB. Media with higher pH were not tested, because CU is known to be unstable in alkaline media and degrade to ferulic acid derivatives [52,53]. Repetitive differential pulse voltammograms were recorded on non-activated (PGE_HB) (Figure 2) and activated working electrodes (PGE_HB*) (Figure S2). During the first anodic scan on PGE_HB, the DPV curves presented a large signal, composed of two unresolved signals, a1 and a2 (Figure 2a), while at PGE_HB* only one peak was observed, along a large signal, and at less positive potentials, due to the electrolyte (Figure S2a). With increasing repetitive scans performed on the same working electrode, the CU signals a1 and a2 became better resolved; the intensity of the signal from less positive potentials (a1) increased, while that of signal a2 decreased (Figure 2b and Figure S2b). During the cathodic scan only, a well-defined reduction wave was observed, whose height was enhanced with the increase in the number of scans (Figure 2c and Figure S2c). However, it must be emphasized that in the cathodic scan, at the activated electrode, the supporting electrolyte also presented a signal at less anodic potential. Regardless of the solution pH and whether the PGE was pretreated or not, the amplitude of both the anodic (a1) and cathodic (c1) signals increased until the 5th–6th scan.

- The influence of the nature of the supporting electrolyte:

For the first anodic scan, the highest DPV signal of CU at both the non-pretreated and the electroactivated PGEs was recorded in BRB pH 6.80, but it was an unresolved peak and a very large one, respectively. The cathodic signal and the well-defined anodic peak a1 obtained after repetitive potential scans at both the PGE_HB and the PHE_HB* were highest in the BRB solution with pH 1.81. Therefore, the anodic and cathodic peak intensities obtained in the 6th scan at PGE_HB and PGE_HB* for CU in several acidic supporting electrolytes were compared (Figure S3). The most intense DPV signal (1.54 × 10^−5^ A) was observed for CU reduction at PGE_HB in 0.05 mol × L^−1^ H_2_SO_4_, while that obtained in 0.10 mol × L^−1^ HNO_3_ at the same type of electrode was slightly lower (1.48 × 10^−5^ A). Even though both the anodic and cathodic peaks on PGE_HB* were higher when 0.10 mol × L^−1^ HNO_3_ was used as the supporting electrolyte, they were less intense than the CU reduction peak recorded at PGE_HB in 0.05 mol × L^−1^ H_2_SO_4_. Therefore, these conditions were further applied for the voltammetric investigation of CU.

### 3.2. Voltammetric Investigation of CU at Disposable PGEs

#### 3.2.1. The Effect of the Solution pH

As can be observed from Figure 2 and Figure S2, the voltammetric behavior of CU at the non-activated and electroactivated PGEs, respectively, was influenced by the pH of the supporting electrolyte, with both the peak height and potential varying with the pH. The shift of the peaks towards lower potentials with increasing pH values indicated that the electrode processes involved protons as well as electrons. Comparing the slopes of the different E_p_ = f (pH) dependencies (Table S1) with the theoretical value from the Nernst equation ($E=E^{0\prime }-\frac{0.059z}{n}pH$, where z and n represent the number of protons and electrons involved in the redox process, respectively, and $E^{0\prime }$ is the corresponding formal redox potential), one can conclude that the ratio z/n was approximately 1 in the anodic processes as well as the cathodic one. The obtained results were in accordance with previously published data [54,55,56,57,58,59].

#### 3.2.2. The Effect of the Potential Scan Rate

The electrode processes of CU at PGE_HB were investigated by CV in 0.05 mol × L^−1^ H_2_SO_4_ as the supporting electrolyte, performing six potential cycles on each pencil lead (Figure S4). It was observed that, at the first potential cycle at PGE_HB, the voltammetric curve showed two anodic waves (a1 and a2) and a well-defined cathodic peak (c1). Starting with the second voltammetric scan, the anodic wave from less positive potentials (a1) turned into a well-defined oxidation peak, whose intensity was enhanced with increasing numbers of cycles, while wave a2, located at higher potentials, decreased, and starting with the third scan it was no longer observed. The disappearance of this signal and the increase in the height of peaks a1 and c1 suggested that this pair of peaks was due to a redox couple belonging to the oxidation product of CU, generated in the irreversible process corresponding to the wave a2, which, according to the literature, was attributed to CU’s oxidation to *o*-benzoquinone [54,59]. The enhancement of the peak pair a1-c1 with the increasing number of voltammetric potential cycles may suggest the formation of a conductive polymeric film at the working electrode surface [60].

**Figure 3:**
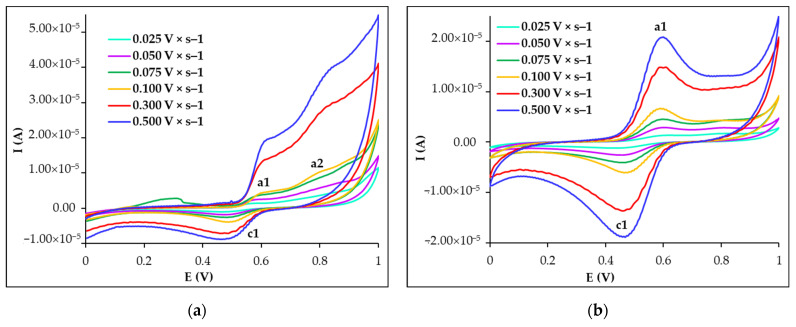
Cyclic voltammograms recorded at PGE_HB at different scan rates for 3.00 × 10^−5^ mol × L^−1^ CU in 0.05 mol × L^−1^ H_2_SO_4_: (**a**) 1st scan and (**b**) 6th scan.

Regardless of the number of potential cycles, the peak current increased with increasing scan rate, while the peak potential remained almost constant for all voltammetric peaks of CU recorded by CV at PGE_HB (Figure 3). The ratio of the anodic and cathodic peak currents (I_pa1_/I_pc1_) measured in the sixth potential scan was almost 1, indicating that the a1-c1 peak pair corresponded to an almost reversible electron donor–acceptor pair, which, based on the literature data, was ascribed to the *o*-benzoquinone–hydroquinone redox couple [54]. Based on similar results, the mechanism for CU’s electrode process was already proposed in various previously published papers [54,56,59,61].

To elucidate the nature of the electrode process of CU at PGE_HB in 0.05 mol × L^−1^ H_2_SO_4_ medium, the different dependences of the peak currents on the scan rate for the first and the sixth potential cycles were analyzed (Table 2). It is known that, for a process controlled by the analyte’s diffusion towards the electrode surface, the peak current depends linearly on the square root of the scan rate, and the slope of the log I_p_—log v dependence is close to 0.5. In contrast, for a surface-confined electrode process, the I_p_ = f(v) relation is linear, and the log I_p_ = f(log v) exhibits a slope close to 1. Analyzing the different dependencies of CU’s peak currents on the potential scan rate (Table 2), it can be concluded that peak a2, which was observed only in the first voltammetric cycles, was determined by CU’s adsorption on the PGE_HB surface. Even though the signals corresponding to the c1/a1 redox peak pair had a diffusion-limiting component in the first scan, they also corresponded to an adsorption-controlled electrode process of CU, as was previously reported for some modified electrodes like β-CD–rGO/GCE [55], pTY/CPE [56], Fe-WO_3_/CPE [59], and SDS/CNPE [61].

### 3.3. Voltammetric Quantification of CU at Disposable PGEs

**Figure 4:**
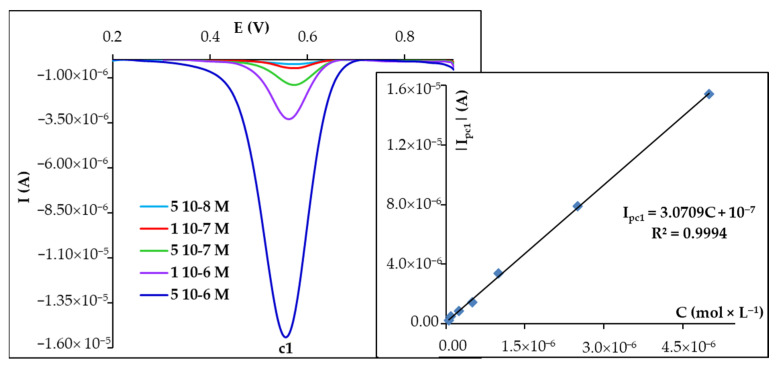
Differential pulse voltammograms (selection) recorded during the 6th scan at PGE_HB for solutions of different CU concentrations in 0.05 mol × L^−1^ H_2_SO_4_. Inset: the dependence of I_pc1_ on CU concentration.

Since the highest voltammetric signal of CU was its reduction peak (c1) recorded at the sixth DPV scan at PGE_HB in 0.05 mol × L^−1^ H_2_SO_4_ (Figure S3), this was further exploited for the quantification of this antioxidant. The influence of the analyte concentration varied from 5.00 × 10^−8^ to 5.00 × 10^−6^ mol × L^−1^ CU, and the variation of the maximum height of this peak (c1) was investigated (Figure 4). A linear dependence between the intensity of this peak (I_pc1_) and the analyte concentration was observed for the entire concentration range tested.

The limits of detection (LOD) and quantification (LOQ) were calculated as 3.3 × σ/S and 10 × σ/S, respectively, where σ represents the standard deviation of the signal measured for the concentration corresponding to the lowest value of the linear range, and S stands for the slope of the calibration graph. The obtained LOD and LOQ values were 2.12 × 10^−8^ mol × L^−1^ CU and 6.42 × 10^−8^ mol × L^−1^ CU, respectively.

Compared to the performance characteristics of other electrochemical sensors published in the literature for CU quantification (Table 3), PGE_HB presents a range of linearity (two orders of magnitude) and an LOD (at the 10^−8^ mol × L^−1^ level) similar to or even better than those of other electrodes, even when modified with different (nano)materials.

The repeatability of the cathodic response of CU at PGE_HB, expressed as the relative standard deviation (RSD%) assessed for 5.00 × 10^−8^ mol × L^−1^ CU (0.018 ppm) and 5.00 × 10^−7^ mol × L^−1^ CU (0.184 ppm), considering the peak current recorded in the sixth scan at five different pencil leads, was 9.11% and 4.17%, respectively. These values were within the accepted limits, which indicate an RSD% value of 11 for an analyte concentration of 1 ppm [76].

### 3.4. Analytical Application of PGE_HB to CU Quantification in Real Samples

**Figure 5:**
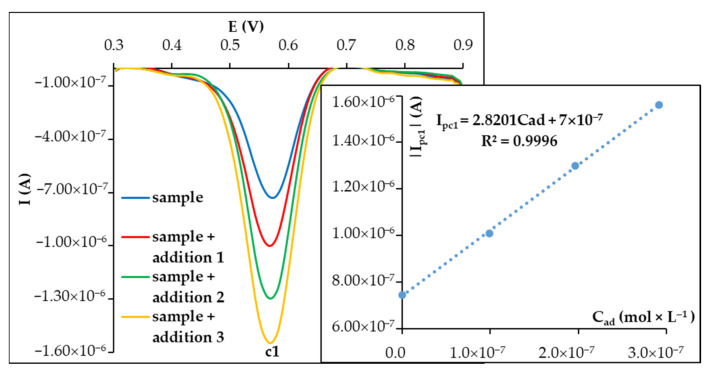
Differential pulse voltammograms recorded during the 6th scan at PGE_HB for a solution of dietary supplement in 0.05 mol × L^−1^ H_2_SO_4_ before and after each addition of 0.10 mL of 1.00 × 10^−5^ mol × L^−1^ CU solution. Inset: the dependence of I_pc1_ on the concentration of added CU.

The analytical application of the developed DPV method at PGE_HB was tested by assessment of the CU content of dietary supplement capsules. The sample solution of dietary capsules was prepared as previously described in Section 2. The multiple standard addition method was applied. The cathodic peak currents (I_pc1_) measured at PGE_HB during the sixth scan for the sample solution, before and after each of the three standard additions of CU stock solution (Figure 5), were used to calculate the CU of the analyzed preparation. The DPV curves of the diluted sample solution presented only a reduction signal with the peak potential characteristic for CU. This signal increased with the addition of the CU standard solution. The CU content of the dietary supplement capsules calculated from the experimental data, considering the mass of the sample taken in the analysis and the dilutions carried out, was compared with that claimed by the manufacturer. The analysis was performed in triplicate. An average recovery of 99.28% with an RSD% of 2.04% was obtained.

### 3.5. Theoretical Calculations

In aqueous acidic solutions at low pH values, CU predominates in its β-diketone form [1,77], fully protonated [78], as depicted in Figure 1.

We focused our attention on the conformational analysis of the β-diketone form to search for the most stable, global minimum conformer, using freshly developed computational tools: the global optimizer algorithm implemented in the newly released (25 July 2024) Orca 6 software. The calculation was performed at the GNF2-xTB level, with an additional recent implementation: the accurate treatment of solvation effects by a continuum solvation model (CPCM-X) [79]. We retained the first four conformers found (in decreasing order of stability), which accounted for about 60% of the overall conformer population (Table S2). The geometry of each of the four conformers retained was refined by subsequent optimizations at the DFT level (initially B3LYP—def2-SVP followed by B3LYP—def2-TZVP) to check whether the conformers found from the preliminary xTB calculation were indeed distinct. All four geometries were distinct, being characterized by very small Gibbs energy differences between them. All final geometries presented a folded shape, where the planes of the two aromatic rings were stacked at around 3.5 Å distance, thus suggesting a possible stabilization effect by π–π stacking interactions. This hypothesis may be explained by taking into account all calculations performed, including the treatment of dispersion correction that accounts for weak noncovalent interactions. All calculations performed include the treatment of the dispersion correction, that accounts for weak non-covalent interactions. Thus, considering the dispersion correction is very important and affects both the stereochemistry and the energetics of the CU molecule, as also found by Madinah et al., who also obtained a similarly folded β-diketone geometry (conformer noted as k8 from ref [80].

An additional factor that can contribute to the stabilization of the folded shape of CU β−diketone conformers in water solution is the formation of intramolecular hydrogen bonds with water molecules. We studied this effect using the geometry of conformer 1 with the *SOLVATOR* algorithm from Orca 6, which attempts explicit solvation by successively adding and docking water molecules to the solute. Of the generated structures, by subsequent treatment at the DFT level, we kept only one, accounting for a single water molecule interacting with both phenolic -OH moieties of the CU molecule via hydrogen bonds (Figure S5). This interaction stabilizes the energy of the complex with 4.62 kcal/mol—a value calculated with B3LYP, def2-TZVP, and CPCM (water). Our further attempts to dock one water molecule to the oxygen atoms from the β-diketone structure and replicate a previously reported geometry [80] did not succeeded due to the large distance between our two carbonyl oxygen atoms (~3.64 Å).

**Figure 6:**
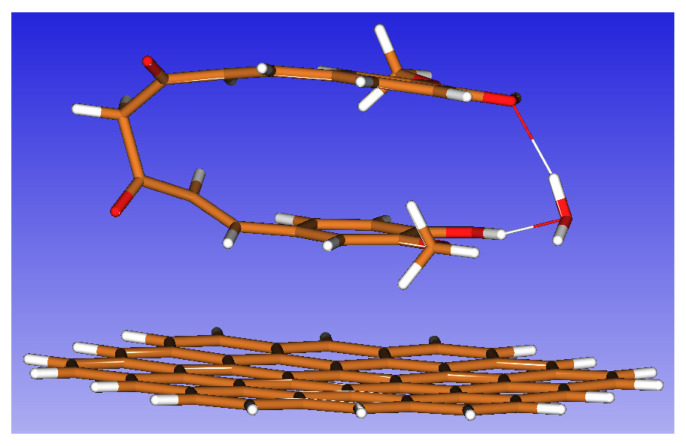
B3LYP def2-TZVP geometry for the interaction between the CU–water complex and circumcoronene. The distance between the stacked aromatic ring plane of CU and the circumcoronene surface is ~3.26 Å.

Another consequence arising from the stabilized folded geometry of the CU β-diketone conformer may be a preferred orientation when interacting with the graphite electrode surface. Coronene is a reduced-scale model of choice for theoretical calculations simulating the interactions of small molecules with the graphene/graphite surface [81]. Our preliminary attempts to dock the CU molecule on coronene did not succeed due to the limited size of the coronene molecule, too small to properly accommodate the CU conformer on its surface. Despite the significant increase in the computational DFT effort, the solution consists in increasing the size of the polyaromatic hydrocarbon (PAH) from coronene to circumcoronene—a recently synthesized molecule [82] that ceases to be only a theoretical graphene/graphite surrogate. The full DFT optimization and Hessian calculation at the B3LYP—def2-TZVP CPCM (water) level of the complex between the hydrated β−diketone conformer 1 of CU (presented in Figure S5) and the circumcoronene surface induces a noncovalent stacking interaction with an associated stabilization energy of 6.92 kcal/mol (Figure 6), where the distance between the plane of the benzene ring of CU and the circumcoronene surface is 3.26 Å. Despite the simplicity of the non-periodic treatment of the graphite surface (assimilated as circumcoronene), this result suggests the geometry of the water-solution adsorption complex between the CU and the surface of the graphite electrode. It is noteworthy that the calculated geometry may explain why the adsorption limits the electrochemical redox processes to occur at the electroactive moieties of only one of the benzene ring.

**Figure 7:**
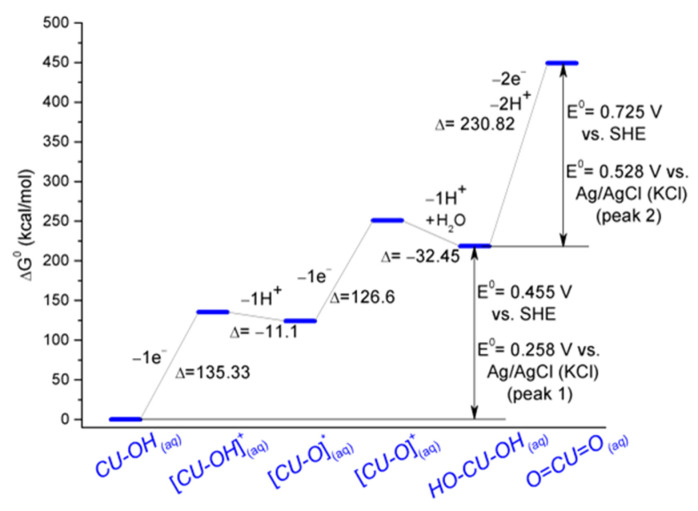
Profile of Gibbs energy variation for the oxidation processes of CU calculated starting from conformer 1, according to the mechanism described in Section 3.2.

Modeling the energetics of the electrochemical oxidation of CU based on the mechanism described in Section 3.2 was the next objective of our theoretical study. Starting from the folded β-diketone structure of conformer 1 (hereafter denoted as CU-OH, to highlight its electroactive phenolic -OH moiety), a process of oxidation by the removal of one electron was performed, leading to the noted intermediate [CU-OH]^+^, which transforms into the free radical [CU-O]· by expelling one proton. The subsequent removal of a second electron leads to the next cationic intermediate [CU-O]^+^, which reacts with one water molecule, freeing a second proton. The resulting product is a stable molecule, denoted as HO-CU-OH, which accounts for the catechol-like structure where a second phenolic -OH substituent is introduced in the aromatic ring at the free *ortho* position of the already-existing phenolic -OH group. By two-electron, two-proton transfer, this molecule can be reversibly oxidized to another stable compound featuring an *o*-benzoquinone structure, denoted as O=CU=O. Each of the abovementioned structures was first optimized at the DFT level (B3LYP def2-TZVP), while taking into consideration the water solvation effects by the CPCM model. Hessian calculations were also performed to assess the thermochemical properties. To obtain more accurate values of the Gibbs energy, subsequent higher-level coupled-cluster single-point calculations in solution (DLPNO CCSD(T) with an extended def2-QZVPP basis set, CPCM) were performed on the previously optimized geometries to re-evaluate the electronic energy. These new values of the electronic energy served as a basis to recalculate more accurate values of the Gibbs energy, allowing us to determine the free energy variation for each process. The results are presented in Figure 7: the ΔG variations were calculated taking into account a molar free energy value of −11.72 eV for the solvated proton [83,84] and a contribution of −0.868 kcal/mol for the gas-phase electron [85]. Taking into account the stable molecules from the diagram, two main electrochemical processes can occur: a first oxidation process (CU-OH → HO-CU-OH) and a second *o*-benzoquinone–hydroquinone redox couple (HO-CU-OH ↔ O=CU=O). The ΔG values of these last two processes allow for the calculation of the theoretical reduction potential E^0^_O|R_ that can be referenced vs. SHE, considering an absolute value of ΔG^0^_SHE_ = −4.28 eV for the free Gibbs energy change of SHE [86,87]. The E^0^_O|R_ value obtained vs. SHE was finally recalculated vs. the Ag/AgCl (saturated KCl) reference electrode, thus predicting the theoretical values of E^0^_O|R_ = 0.258 V and 0.528 V, respectively, for the abovementioned electrochemical processes.

## 4. Conclusions

CU is a natural polyphenolic compound with multiple applications in the medicine, food, and cosmetics industries, due to its well-known biological activities, especially its antioxidant properties. Due to the presence of the phenolic hydroxyl groups in its molecule, CU is electroactive and can be analysed by voltammetry. This work discusses CU’s redox behavior at the disposable PGE for the first time. In the first anodic scan, CU presented a large oxidation signal composed of two unresolved peaks. However, in the cathodic scan and in the subsequent anodic scans, CU showed a pair of well-defined redox peaks whose intensity was enhanced with the increase in the number of successive scans to six. These pH-dependent and adsorption-controlled peaks were assigned to the *o*-benzoquinone–catechol couple.

Theoretical calculations indicate that the most stable fully protonated β-diketone CU conformers present in acidic water solutions feature a folded structure with stacked aromatic rings, stabilized by noncovalent π–π interactions and intramolecular hydrogen bonds with a water molecule. From the full-DFT calculations, this conformation was shown to interact with the surface of the graphite electrode, causing the electroactive moieties attached to only one of the two aromatic rings of CU to undergo redox processes. Accurate calculation of the electronic energy by the coupled-clusters method allowed for the correction of the free Gibbs energy of the structures involved in the electrochemical oxidation processes. Theoretical values of the reduction potential were calculated for both of the experimentally observed CV peaks.

Studies for the optimization of the working conditions revealed that the best-defined and highest voltammetric signal of CU was the reduction signal obtained during the sixth cathodic scan at PGE_HB, using 0.05 mol × L^−1^ H_2_SO_4_ as the supporting electrolyte. This signal increased linearly with the analyte concentration in the range 5.00 × 10^−8^–5.00 × 10^−6^ mol × L^−1^ CU, enabling the development of a DPV method for CU quantification, with a linear range of two orders of magnitude, an LOD of 2.12 × 10^−8^ mol × L^−1^ CU, and repeatability within the accepted limits. These are the best performance characteristics attained for CU’s voltammetric quantitative determination using a non-modified working electrode. However, there are a few reports of modified working electrodes that presented lower LODs [57,71,72,73,74] for this analyte, but these involved time- and reagent-consuming preparation steps. The main advantage of the DPV method described here for CU analysis is the employment of a cheap, eco-friendly, disposable, and commonly commercially available PGE_HB. The method can be used for rapid, simple, and sensitive CU screening, with application in the quality control of CU in dietary supplements.

## Figures and Tables

**Table 1:** CU anodic peak potentials (E_pa_, V) recorded by DPV at different types of working electrodes, and the corresponding sensitivities (S, A × L × mol^−1^ × cm^−2^). Supporting electrolyte: ABS pH 3.50.

Working Electrode	E_pa_ (V)	S (A × L × mol^−1^ × cm^−2^)
GCE	0.674	3.793
PGE_2B	0.664	4.189
PGE_B	0.674	4.264
PGE_HB	0.669	4.766
PGE_H	0.649	2.283

**Table 2:** Different dependencies of peak current on scan rate.

Scan	I_p_ = f (v)	I_p_ = f (v^1/2^)	log I_p_ = log (v)
1	I_pa1_ = 1 × 10^−5^ × v + 1 × 10^−5^; R^2^ = 0.9987	I_pa1_ = 1 × 10^−5^ × v^1/2^ − 2 × 10^−6^; R^2^ = 0.9706	log I_pa1_ = 0.8861 × log v– 4.8829; R^2^ = 0.9916
	I_pa2_ = 7 × 10^−6^ × v − 3 × 10^−8^; R^2^ = 0.9635	I_pa2_ = 6 × 10^−6^ × v^1/2^ − 1 × 10^−6^; R^2^ = 0.9760	log I_pa2_ = 1.2166 × log v − 5.0276; R^2^ = 0.9900
	I_pc1_ = –1 × 10^−5^ × v − 1 × 10^−6^; R^2^ = 0.9333	I_pc1_ = –1 × 10^−5^ × v^1/2^ + 7 × 10^−7^; R^2^ = 0.9880	log I_pc1_ = 0.7017 × log v − 4.9031; R^2^ = 0.9748
6	I_pa1_ = 3 × 10^−5^ × v + 4 × 10^−7^; R^2^ = 0.9842	I_pa1_ = 3 × 10^−5^ × v^1/2 −^ 4 × 10^−6^; R^2^ = 0.9936	log I_pa1_ = 1.0937 × log v– 4.4303; R^2^ = 0.9665
	I_pc1_ = –3 × 10^−5^ × v − 1 × 10^−6^; R^2^ = 0.9805	I_pc1_ = –3 × 10^−5^ × v^1/2^ + 4 × 10^−6^; R^2^ = 0.9969	log I_pc1_ = 0.9541 × log v − 4.4373; R^2^ = 0.9768

**Table 3:** Performance characteristics of electrochemical sensors applied to CU quantification.

Electrode	Technique	Working Conditions	Linear Range (mol × L^−1^)	LOD (mol × L^−1^)	Sample	Reference
MIP/CPE	CV + MDA	PBS pH 6.00	1.00 × 10^−6^–1.00 × 10^−4^		Turmeric powder	[62]
HaP-IL/PGE	DPV	ABS pH 4.80 t_acc_ = 5 min	5.43 × 10^−6^–2.71 × 10^−5^	5.04 × 10^−6^	CU_DNA interaction	[27]
p(Van-CA)/Pt	DPV	PBS pH 7.25	1.00 × 10^−5^–1.00 × 10^−3^	5.00 × 10^−6^	Spices	[63]
pTY/CPE	CV	PBS pH 6.50	2.00 × 10^−6^–1.00 × 10^−5^ 1.00 × 10^−5^–4.00 × 10^−5^	1.09 × 10^−6^	Food supplement	[56]
CPE in 0.01 mol/L β-CD	CV	ABS pH 3.57	2.50 × 10^−6^–2.70 × 10^−5^	9.30 × 10^−7^	Turmeric spice	[64]
MWCNTs /BPPGE	AdS-CV	BRB pH 1.81 t_acc_ = 60 s	2.00 × 10^−6^–1.00 × 10^−4^	4.50 × 10^−7^	Turmeric powder	[54]
EPPGE	DPV	0.1 mol × L^−1^ KCl + HCl pH 2.00	3.25 × 10^−7^–1.95 × 10^−6^	2.96 × 10^−7^	Turmeric powder	[65]
Fe-WO_3_/CPE	SWV	PBS pH 3.00	5.00 × 10^−6^–6.00 × 10^−5^	6.20 × 10^−8^	Turmeric powder	[59]
pACBK/GCE	DPV	PBS pH 6.40 t_acc_ = 70 s	1.00 × 10^−7^–7.00 × 10^−5^	4.10 × 10^−8^	Human urine	[66]
pAA_MIP /GE	DPV	ABS pH 5.50	1.00 × 10^−6^–1.00 × 10^−5^ 1.00 × 10^−5^–1.80 × 10^−4^	4.00 × 10^−8^	Raw turmeric, turmeric powder, capsule	[67]
MIP_MWCNTs/GCE	DPV	BRB pH 2.00	4.00 × 10^−8^–2.00 × 10^−6^ 2.00 × 10^−6^–4.00 × 10^−5^	3.70 × 10^−8^	Capsule, plant root, food seasoning	[68]
β-CD–rGO/GCE	DPV	PBS pH 7.00 t_acc_ = 45 min	5.00 × 10^−8^–1.00 × 10^−5^	3.30 × 10^−8^	-	[55]
Gr/GCE	LSV	0.10 mol × L^−1^ H_2_SO_4_	5.00 × 10^−8^–3.00 × 10^−6^	3.00 × 10^−8^	*Curcuma longa L.*	[68]
pCys_MIP-CuCo_2_O_4_-*N*-CNTs-P-GO/GCE	DPV	PBS pH 3.06	1.00 × 10^−7^–1.00 × 10^−6^ 1.00 × 10^−6^–3.00 × 10^−5^	3.00 × 10^−−8^	Human blood serum	[69]
pGA/CNTsPE	DPV	PBS pH 7.50	4.00 × 10^−7^–6.00 × 10^−6^ 6.00 × 10^−6^–1.00 × 10^−5^	2.79 × 10^−8^	Food supplement	[70]
SDS/CNPE	DPV	PBS pH 6.00	2.00 × 10^−7^–1.00 × 10^−6^ 1.50 × 10^−6^–4.50 × 10^−6^	2.70 × 10^−8^	Food supplement	[61]
MnO_2_-c-MWCNTs /GCE	SDLSV	0.10 mol × L^−1^ H_2_SO_4_ E_acc_ = −0.300 V; t_acc_ = 90 s	1.00 × 10^−8^–1.00 × 10^−6^ 1.00 × 10^−6^–8.00 × 10^−5^	6.00 × 10^−9^	Turmeric powder, curry, mustard, instant noodle seasoning, ginger powder	[57]
MWCNTs/GCE Dy_NWs/CPE	SWV FFTSWV	PBS pH 4.00 E_acc_ = −0.700 V; t_acc,SWV_ = 110 s; t_acc,FFTSWV_ = 0.4 s	1.00 × 10^−8^–1.00 × 10^−6^ 2.00 × 10^−9^–1.00 × 10^−6^	5.00 × 10^−9^ 5.00 × 10^−10^	Milk	[71]
Ce-BDC-MOF_NPs/GPE	SWASV	BRB pH 3.00 E_acc_ = 0.100 V; t_acc_ = 40 s	2.00 × 10^−11^–2.00 × 10^−9^ 2.00 × 10^−9^–9.00 × 10^−9^ 5.00 × 10^−11^–7.00 × 10^−9^ 3.00 × 10^−11^–6.00 × 10^−9^	6.00 × 10^−12^ 1.50 × 10^−11^ 9.00 × 10^−12^	Bulk Human plasma Human urine	[72]
GCE + 10 ppm SDS	SWAdSV	0.20 mol × L^−1^ CH_3_COOH E_acc_ = 0.000 V; t_acc_ = 120 s	1.00 × 10^−12^–2.00 × 10^−8^	2.70 × 10^−13^	Spice extracts, energy shots	[73]
NSrGO/Ru@AuNPs/GCE	SWV	PBS pH 5.00	1.00 × 10^−12^–1.00 × 10^−10^	2.00 × 10^−13^	Plasma	[74]
CPE	DPV	PBS pH 3.00	3.00 × 10^−6^–3.00 × 10^−4^	5.03 × 10^−6^	Human blood serum	[75]
PGE	DPV	0.05 mol × L^−1^ H_2_SO_4_	5.00 × 10^−8^–5.00 × 10^−6^	2.12 × 10^−8^	Dietary supplements	This work

## Data Availability

Data are contained within the article.

## Supplementary Materials

The following supporting information can be downloaded at: https://www.mdpi.com/article/10.3390/bios15030137/s1, Figure S1. Differential pulse voltammograms recorded for 4.00 × 10^−6^ mol × L^−1^ CU in ABS pH 3.50, at PGE not activated (PGE_HB) and electrochemically pretreated (PGE_HB*) in different conditions, respectively; Figure S2. Differential pulse voltammograms recorded at PGE_HB* for 5.00 × 10^−6^ mol × L^−1^ CU in BRB solutions with different pH values in: (a) anodic direction—scan 1; (b) anodic direction—scan 6 and (c) cathodic direction—scan 1 and scan 6; Figure S3. DPV peak currents recorded at the 6^th^ scan for 5.00 × 10^−6^ mol × L^−1^ CU in different acidic supporting electrolytes, at PGE_HB and PGE_HB*, respectively; Figure S4. Repetitive cyclic voltammograms recorded at PGE_HB for 3.00 × 10^−6^ mol × L^−1^ CU in 0.05 mol × L^−1^ H_2_SO_4_, scan rate 0.100 V × s^−1^; Figure S5. Stabilization of CU conformer 1 geometry by explicit solvation. The water molecule attaches through intramolecular hydrogen bonds to the phenolic -OH groups; Table S1. The E_p_ = f(pH) dependencies for CU voltammetric peaks recorded at disposable PGE; Table S2. Most stable conformers of CU β-diketone form in water solution: conformer geometry, relative energy difference and percentage from the overall conformers population (both calculated at xTB CPCM-X level), superposition between the geometries optimized at xTB CPCM-X (red) and B3LYP-def2-TZVP CPCM (cyan) levels; relative Gibbs energy difference (from B3LYP-def2-TZVP CPCM Hessian calculations).

## Abbreviations

The following abbreviations are used in this manuscript:

ABS	Acetate Buffer Solution
AdS-CV	Adsorptive Stripping Cyclic Voltammetry
β-CD	β-Cyclodextrin
β-CD–RGO/GCE	β-Cyclodextrin-reduced Graphene-Oxide-Modified Glassy Carbon Electrode
B3LYP	Becke three-parameter Lee–Yang–Parr hybrid density functional
BRB	Britton–Robinson Buffer
CCSD(T)	Coupled-Cluster Singles, Doubles, and Perturbative Triples
Ce-BDC-MOF_NPs/GPE	Ce-1,4-Benzenedicarboxylic Metal–Organic Framework Nanoparticle-Modified Graphite Paste Electrode
CPCM	Conductor-Like Polarizable Continuum Model for Solvation Effects
CPCM-X	Extended Conductor-Like Polarizable Continuum Solvation Model
CPE	Carbon Paste Electrode
C-SPE	Carbon Screen-Printed Electrode
CU	Curcumin
CV	Cyclic voltammetry
DFT	Density Functional Theory
DLPNO	Domain-based local pair natural orbital
DPV	Differential pulse voltammetry
Dy_NWs/CPE	Dysprosium Nanowire-Modified Carbon Paste Electrode
E_acc_	Accumulation potential
EPPGE	Edge Plane Pyrolytic Graphite Electrode
Fe-WO_3_/CPE	Iron-Doped Tungsten-Oxide-Modified Carbon Paste Electrode
FFTSWV	Fast Fourier Transform Square-Wave Voltammetry
FT-IR	Fourier-Transform Infrared
GCE	Glassy Carbon Electrode
GC-MS	Gas Chromatography–Mass Spectrometry
GFN	Geometry, Frequency, and Noncovalent Interactions
Gr/GCE	Graphene-Modified Glassy Carbon Electrode
HaP-IL/PGE	Hydroxyapatite Nanoparticles and Ionic-Liquid-Modified Pencil Graphite Electrode
HPTLC	High-Performance Thin-Layer Chromatography
LOD	Limit of detection
LSV	Linear Sweep Voltammetry
MDA	Multivariate Data Analysis
MIP/CPE	Molecularly Imprinted Polymer-Modified Carbon Paste Electrode
MIP_MWCNTs/GCE	Molecularly Imprinted Polymer/Multiwalled Carbon Nanotube-Modified Glassy Carbon Electrode
MnO_2_-c-MWCNTs/GCE	MnO_2_ Nanoparticle-Functionalized Carboxylated Multiwalled Carbon Nanotube-Modified Glassy Carbon Electrode
MoS_2_ QDs	MoS_2_ Quantum Dots
MWCNTs/BPPGE	Multiwalled Carbon Nanotube-Modified Basal Plane Pyrolytic Graphite Electrode
MWCNTs/GCE	Multiwalled Carbon Nanotube-Modified Glassy Carbon Electrode
NCDs	Nitrogen-doped Carbon Dots
NSrGO/Ru@AuNPs/GCE	Ru@Au Nanoparticle-Decorated Nitrogen- and Sulfur-Functionalized Reduced Graphene Oxide-Modified Glassy Carbon Electrode
pAA_MIP/GE	Polyacrylic Acid-Based Molecularly Imprinted Polymer-Embedded Graphite Electrode
pACBK/GCE	Poly(Acid Chrome Blue K)-Modified Glassy Carbon Electrode
PBS	Phosphate Buffer Solution
PCR	Polymerase Chain Reaction
pCys_MIP-CuCo_2_O_4_-*N*-CNTs-P-GO/GCE	Poly(L-Cysteine)-Based Molecularly Imprinted Polymer-CuCo_2_O_4_-Nitrogen-Doped Carbon Nanotubes-Phosphorus-Doped Graphene-Oxide-Modified Glassy Carbon Electrode
pGA/CNTsPE	Poly(Glutamine) Film-Modified Carbon Nanotubes Paste Electrode
PGE	Pencil graphite electrode
pTY/CPE	Poly(Titan Yellow)-Modified Carbon Paste Electrode
p(Van-CA)/Pt	Poly(Vanillin-co-Caffeic Acid)-Modified Platinum Electrode
QZVPP	Valence Quadruple-Zeta with Two Sets of Polarization Functions Basis Set
RP_HPLC	Reversed-Phase High-Performance Liquid Chromatography
SDLSV	Second-Order Derivative Linear Sweep Voltammetry
SDS	Sodium Dodecyl Sulfate
SDS/CNPE	Sodium Dodecyl Sulfate-Modified Carbon Nanocomposite Paste Electrode
SHE	Standard Hydrogen Electrode
SVP	Split-Valence Polarized Basis Set
SWAS	Square-Wave Anodic Stripping Voltammetry
SWV	Square-Wave Voltammetry
t_acc_	Accumulation time
TZVP	Triple-Zeta Valence with Polarization Basis Set
WHO	World Health Organization
xTB	Extended Tight-Binding Quantum Chemistry Methods

## References

[1] David I.G., Iorgulescu E.E., Popa D.E., Buleandra M., Cheregi M.C., Noor H. (2023). Curcumin Electrochemistry—Antioxidant Activity Assessment, Voltammetric Behavior and Quantitative Determination, Applications as Electrode Modifier. Antioxidants.

[2] da Silva Gündel S., Zorzanello B., Reis Favarin F., Forrati Machado É., Schafer da Silva A., da Silva W.L., Dal Pont Morisso F., Ferreira Ourique A. (2025). Redispersible dry powders containing nanoencapsulated curcumin increase its antioxidant activity. J. Food Eng..

[3] Xu T., Yan L., Liu C., Zheng L. (2025). Novel functional carriers based on Spirulina protein for the delivery of curcumin with improved stability and antioxidant efficiency. Food Chem..

[4] Shariare M.H., Mannan M., Khan F., Sharmin A., Attwa M.W., Rahman A.F.M.M., Rahman M., Uddin M.N., Kazi M. (2024). Phospholipid-based nano drug delivery system of curcumin using MSP1D1 protein and poloxamer 407: A comparative study for targeted drug delivery to the brain. J. Nanopart. Res..

[5] Roy S., Priyadarshi R., Ezati P., Rhim J.-W. (2022). Curcumin and its uses in active and smart food packaging applications—A comprehensive review. Food Chem..

[6] Pourasgar S., Ranji N., Asadpour L., Shahriarinour M., Nikpassand M. (2024). Antibacterial and Anti-cancer Properties of Curcumin-Functionalized Silica-Coated Fe_3_O_4_ Magnetic Nanoparticles. Arab. J. Sci. Eng..

[7] Sayyar Z., Jafarizadeh-Malmiri H. (2024). Enhancing the efficacy of nano-curcumin on cancer cells through mixture design optimization of three emulsifiers. BMC Chem..

[8] Asadi S., Madrakian T., Ahmadi M., Aguirre Á.M., Afhami A., Uroomiye S.S., Ghafari F., Ranjbar A. (2023). Aerosol assisted synthesis of a pH responsive curcumin anticancer drug nanocarrier using chitosan and alginate natural polymers. Sci. Rep..

[9] Deng W., Xiong X., Lu M., Huang S., Luo Y., Wang Y., Ying Y. (2024). Curcumin suppresses colorectal tumorigenesis through restoring the gut microbiota and metabolites. BMC Cancer.

[10] Soni V.K., Mehta A., Ratre Y.K., Kumar C., Singh R.P., Srivastava A.K., Chaturvedi N., Shukla D., Pandey S.K., Vishvakarma N.K., Shukla D., Vishvakarma N.K., Nagaraju G.P. (2022). Antineoplastic Effects of Curcumin Against Colorectal Cancer: Application and Mechanisms. Colon Cancer Diagnosis and Therapy.

[11] Youshia J., Gabal Y.M., Mansour M., Gad H.A., Rai M., Feitosa C.M. (2023). Curcumin-Loaded Nanoparticles in Neurodegenerative Diseases. Curcumin and Neurodegenerative Diseases.

[12] de Oliveira Barbosa C., Neto J.F.C., Rai M., Feitosa C.M. (2023). Turmeric and Alzheimer’s Disease: Therapeutic Effects of Curcuminoids, Curcumin, and Turmerone. Curcumin and Neurodegenerative Diseases.

[13] Nath P., Dey A., Kundu T., Pathak T., Chatterjee M., Roy P., Satapathi S. (2025). Highly fluorescent nitrogen doped carbon dots as analytical probe for sensitive detection of curcumin through smartphone integrated 3D-printed platform: A new horizon in food safety. Spectrochim. Acta A Mol. Biomol. Spectrosc..

[14] Ablak Ö., Altunay N. (2024). Investigation of different solvents for selective, safe and rapid extraction of curcumin from various food and herbal supplement products: Multivariate strategy and assessment of green profile. Microchem. J..

[15] Salamat Q., Soylak M. (2024). Novel reusable and switchable deep eutectic solvent for extraction and determination of curcumin in water and food samples. Talanta.

[16] Deepa S., Madhu S., Devasenan S., Murali G., Pancharatna P.T., Maaza M., Kaviyarasu K., Jeyaram S. (2024). Extraction of Natural Pigment *Curcumin* from *Curcuma longa*: Spectral, DFT, Third-order Nonlinear Optical and Optical Limiting Study. J. Fluoresc..

[17] Singh A., Avupati V.R. (2017). Development and Validation of UV-Spectrophotometric method for the Estimation of Curcumin in Standardised Polyherbal Formulations. J. Young Pharm..

[18] Jantra J., Teepoo S., Thananimit S. (2024). Smartphone-based imaging colorimetric assay for monitoring the quality of curcumin in turmeric powder. Anal. Sci..

[19] Wang S., Wang X., Ning K., Xiang G. (2024). Fluorescent Molybdenum Disulfide Quantum Dots for Sensitive Detecting Curcumin in Food Samples through FRET Mechanism. J. Fluoresc..

[20] Ge J., Zhai Z., Chen Y., Li Z., Yang H., Cai R. (2024). Nitrogen-doped MoS_2_ QDs as fluorescent probes for sensitive detection of curcumin and cell imaging. Anal. Chim. Acta.

[21] Naz Z., Faisal M.S., Khan A.B., Naz A., Ahmad F.J. (2024). Development of a Validated RP-HPLC Method for the Estimation of Curcumin in Nanoemulsion and in Its Phase Solubility Studies. J. Appl. Spectrosc..

[22] Khairnar A., Lohidasan S., Dubey R., Sankaran S. (2023). Analytical method development and validation for the simultaneous estimation of quercetin, berberine, rutin and curcumin in a polyherbal formulation using high-performance thin-layer chromatography. JPC J. Planar Chromatogr. Mod. TLC.

[23] Setyaningsih D., Santoso Y.A., Hartini Y.S., Murti Y.B., Hinrichs W.L.J., Patramurti C. (2021). Isocratic high-performance liquid chromatography (HPLC) for simultaneous quantification of curcumin and piperine in a microparticle formulation containing Curcuma longa and Piper nigrum. Heliyon.

[24] Rodriguez E.L., Zhang C., Woolfork A.G., Li Z., Bi C., Kaur H., Juritsch A.F., Moreau R., Hage D.S. (2021). Analysis of curcumin and piperine in biological samples by reversed-phase liquid chromatography with multi-wavelength detection. J. Chromatog. B.

[25] Chiorcea-Paquim A.-M. (2023). Electrochemical Sensing of Curcumin: A Review. Antioxidants.

[26] Mousaabadi K.Z., Ensafi A.A., Hadadzadeh H., Shirani M.P. (2024). Impact of temperature on the binding interaction between dsDNA and curcumin: An electrochemical study. Bioelectrochemistry.

[27] Uca M., Eksin E., Erac Y., Erdem A. (2021). Electrochemical Investigation of Curcumin–DNA Interaction by Using Hydroxyapatite Nanoparticles–Ionic Liquids Based Composite Electrodes. Materials.

[28] David I.G., Litescu S.C., Moraru R., Albu C., Buleandra M., Popa D.E., Riga S., Ciobanu A.M., Noor H. (2022). Electroanalysis of Naringin at Electroactivated Pencil Graphite Electrode for the Assessment of Polyphenolics with Intermediate Antioxidant Power. Antioxidants.

[29] Neese F., Wennmohs F., Becker U., Riplinger C. (2020). The ORCA quantum chemistry program package. J. Chem. Phys..

[30] Neese F. (2022). Software update: The ORCA program system, version 5.0. Wiley Interdiscip. Rev. Comput. Mol. Sci..

[31] Bannwarth C., Caldeweyher E., Ehlert S., Hansen A., Pracht P., Seibert J., Spicher S., Grimme S. (2012). Extended tight-binding quantum chemistry methods. Wiley Interdiscip. Rev. Comput. Mol. Sci..

[32] Bannwarth C., Ehlert S., Grimme S. (2019). GFN2-xTB—An accurate and broadly parametrized self-consistent tight-binding quantum chemical method with multipole electrostatics and density-dependent dispersion contributions. J. Chem. Theory Comput..

[33] Neese F. (2023). The SHARK integral generation and digestion system. J. Comp. Chem..

[34] Caldeweyher E., Ehlert S., Hansen A., Neugebauer H., Spicher S., Bannwarth C., Grimme S. (2019). A generally applicable atomic-charge dependent London dispersion correction. J. Chem. Phys..

[35] Caldeweyher E., Mewes J., Ehlert S., Grimme S. (2020). Extension and evaluation of the D4 London-dispersion model for periodic systems. Phys. Chem. Chem. Phys..

[36] Wittmann L., Gordiy I., Friede M., Helmich-Paris B., Grimme S., Hansen A., Bursch M. (2024). Extension of the D3 and D4 London dispersion corrections to the full Actinides series. Phys. Chem. Chem. Phys..

[37] Weigend F., Ahlrichs R. (2005). Balanced basis sets of split valence, triple zeta valence and quadruple zeta valence quality for H to Rn: Design and assessment of accuracy. Phys. Chem. Chem. Phys..

[38] Helmich-Paris B., de Souza B., Neese F., Izsák R. (2021). An improved chain of spheres for exchange algorithm. J. Chem. Phys..

[39] Neese F. (2003). An improvement of the resolution of the identity approximation for the formation of the Coulomb matrix. J. Comp. Chem..

[40] Bykov D., Petrenko T., Izsak R., Kossmann S., Becker U., Valeev E., Neese F. (2015). Efficient implementation of the analytic second derivatives of Hartree-Fock and hybrid DFT energies: A detailed analysis of different approximations. Mol. Phys..

[41] Garcia-Rates M., Neese F. (2020). Effect of the solute cavity on the solvation energy and its derivatives within the framework of the Gaussian charge scheme. J. Comput. Chem..

[42] Neese F., Hansen A., Liakos D.G. (2009). Efficient and accurate approximations to the local coupled cluster singles doubles method using a truncated pair natural orbital basis. J. Chem. Phys..

[43] Neese F., Wennmohs F., Hansen A. (2009). Efficient and accurate local approximations to coupled-electron pair approaches: An attempt to revive the pair natural orbital method. J. Chem. Phys..

[44] Riplinger C., Neese F. (2013). An efficient and near linear scaling pair natural orbital based local coupled cluster method. J. Chem. Phys..

[45] Riplinger C., Sandhoefer B., Hansen A., Neese F. (2013). Natural triple excitations in local coupled cluster calculations with pair natural orbitals. J. Chem. Phys..

[46] Riplinger C., Pinski P., Becker U., Valeev E.F., Neese F. (2016). Sparse maps—A systematic infrastructure for reduced-scaling electronic structure methods. II. Linear scaling domain based pair natural orbital coupled cluster theory. J. Chem. Phys..

[47] Garcia-Rates M., Becker U., Neese F. (2021). Implicit solvation in domain based pair natural orbital coupled cluster (DLPNO-CCSD) theory. J. Comput. Chem..

[48] Schaftenaar G., Noordik J.H. (2000). Molden: A pre- and post-processing program for molecular and electronic structures. J. Comput. Aided Mol. Des..

[49] Jmol: An Open-Source Java Viewer for Chemical Structures In 3D. http://www.jmol.org/.

[50] David I.G., Buleandra M., Popa D.E., Cheregi M.C., David V., Iorgulescu E.E., Tartareanu G.O. (2022). Recent developments in voltammetric analysis of pharmaceuticals using disposable pencil graphite electrodes. Processes.

[51] Sousa M.C., Buchanan J.W. (2000). Observational Models of Graphite Pencil Materials. Comput. Graph. Forum.

[52] Tonnesen H.H., Karlsen J. (1985). Studies on Curcumin and Curcuminoids. Alkaline Degradation of Curcumin. Z. Lebensm. Unters. Forsch..

[53] Wang Y.-J., Pan M.-H., Cheng A.-L., Lin L.-I., Ho Y.-S., Hsieh C.-Y., Lin J.-K. (1997). Stability of curcumin in buffer solutions and characterization of its degradation products. J. Pharm. Biomed. Anal..

[54] Chaisiwamongkhol K., Ngamchuea K., Batchelor-McAuley C., Compton R.G. (2017). Multiwalled Carbon Nanotube Modified Electrodes for the Adsorptive Stripping Voltammetric Determination and Quantification of Curcumin in Turmeric. Electroanalysis.

[55] Mirzaei B., Zarrabi A., Noorbakhsh A., Amini A., Makvandi P. (2021). A reduced graphene oxide-b-cyclodextrin nanocomposite-based electrode for electrochemical detection of curcumin. RSC Adv..

[56] D’Souza E.S., Manjunatha J.G., Raril C., Tigari G., Arpitha H.J., Shenoy S. (2021). Electro-Polymerized Titan Yellow Modified Carbon Paste Electrode for the Analysis of Curcumin. Surfaces.

[57] Deng P., Wei Y., Li W., Shi S., Zhou C., Li J., Yao L., Ding J., He Q. (2023). A Novel Platform Based on MnO_2_ Nanoparticles and Carboxylated Multi-walled Carbon Nanotubes Composite for Accurate and Rapid Determination of Curcumin in Commercial Food Products. J. Food Compos. Anal..

[58] Jara-Cornejo E., Peña-Bedón E., Torres Moya M., Espinoza-Torres S., Sotomayor M.D.P.T., Picasso G., Tuesta J.C., López R., Khan S. (2024). Electrochemical Analysis of Curcumin in Real Samples Using Intelligent Materials. Polymers.

[59] Malode S.J., Navada N., Shanbhag M.M., Shetti N.P., Alodhayb A.N., Alzahrani K.E., Albrithen H., Assaifan A.K. (2024). Fe-doped WO3-Modified sensor for the improved electrochemical detection of curcumin. Microchem. J..

[60] Devadas B., Rajkumar M., Chen S.-M. (2014). Electropolymerization of Curcumin on Glassy Carbon Electrode and its Electrocatalytic Application for the Voltammetric Determination of Epinephrine and p-Acetoaminophenol. Colloids Surf. B Biointerfaces.

[61] Raril C., Manjunatha J.G., Tigari G. (2020). Low-cost voltammetric sensor based on an anionic surfactant modified carbon nanocomposite material for the rapid determination of curcumin in natural food supplement. Instrum. Sci. Technol..

[62] Kar S., Naskar H., Tudu B., Bandyopadhyay R. (2018). Application of a polytrimethoxysilane based molecularly imprinted polymer (MIP) electrode towards discrimination of different types of turmeric powder. Carbon Sci. Technol..

[63] Burç M., Güngör Ö., Duran S.T. (2020). Voltammetric Determination of Curcumin in Spices using Platinum Electrode Electrochemically Modified with Poly(Vanillin-co-Caffeic Acid). Anal. Bioanal. Electrochem..

[64] Martínez-Guerra J., Palomar-Pardavé M., Romero-Romo M., Corona-Avendaño S., Guzmán-Hernández D.-S., Rojas-Hernández A., Ramírez-Silva M.T. (2022). On the Curcumin and β-Cyclodextrin Interaction in Aqueous Media. Spectrophotometric and Electrochemical Study. ChemElectroChem.

[65] Basmaz G., Öztürk N. (2017). Determination of Curcumin in Turmeric Sample Using Edge Plane Pyrolytic Graphite Electrode. Celal Bayar Univ. J. Sci..

[66] Peng J., Nong K., Cen L. (2012). Electropolymerization of Acid Chrome Blue K on Glassy Carbon Electrode for the Determination of Curcumin. J. Chin. Chem. Soc..

[67] Ahmed A.H.M.T., Naskar H., Banerjee S., Ghatak B., Das N., Tudu B., Bandyopadhyay R. (2022). Electrochemical Sensor Based on Molecularly Imprinted Polymer Embedded Graphite Electrode for Detecting Curcumin. Sens. Actuators A Phys..

[68] Li K., Li Y., Yang L., Wang L., Ye B. (2014). The Electrochemical Characterization of Curcumin and its Selective Detection in Curcuma Using a Graphene-Modified Electrode. Anal. Methods.

[69] Mohammadinejad A., Abouzari-Lotf E., Aleyaghoob G., Rezayi M., Oskuee R.K. (2022). Application of a Transition Metal Oxide/Carbon-Based Nanocomposite for Designing a Molecularly Imprinted Poly (l-Cysteine) Electrochemical Sensor for Curcumin. Food Chem..

[70] Tigari G., Manjunatha J.G. (2020). Poly (glutamine) film-coated carbon nanotube paste electrode for the determination of curcumin with vanillin: An electroanalytical approach. Monatsh. Chem..

[71] Daneshgar P., Norouzi P., Moosavi-Movahedi A.A., Ganjali M.R., Haghshenas E., Dousty F., Farhadi M. (2009). Fabrication of Carbon Nanotube and Dysprosium Nanowire Modified Electrodes as a Sensor for Determination of Curcumin. J. Appl. Electrochem..

[72] Elfiky M., Beltagi A.M., Abuzalat O. (2021). Selective Modified Stripping Voltammetric Sensor Based on Ce-1,4-Benzenedicarboxylic Metal–Organic Frameworks Porous Nanoparticles for Picomolar Detection of Curcumin. J. Electroanal. Chem..

[73] Tyszczuk-Rotko K., Keller A., Gorylewski D., Kozak J., Staniec K., Wójciak M., Sowa I. (2024). Sensitive and selective antioxidant curcumin analysis using anionic surfactant modified glassy carbon electrode. Microchem. J..

[74] Kotan G., Kardas F., Yokus O.A., Akyıldırım O., Saral H., Eren T., Yola M.L., Atar N. (2016). A Novel Determination of Curcumin via Ru@Au Nanoparticle Decorated Nitrogen and Sulfur Functionalized Reduced Graphene Oxide Nanomaterials. Anal. Methods.

[75] Rahimnejad M., Zokhtare R., Moghadamnia A.A., Asghary M. (2018). Fabrication of Electrochemical Curcumin Sensor Based on Carbon Paste Electrode. J. Appl. Chem..

[76] AOAC International Guidelines for Standard Method Performance Requirements. Appendix, F. https://www.aoac.org/wp-content/uploads/2019/08/app_f.pdf.

[77] Bhatia N.K., Kishor S., Katyal N., Gogoi P., Narang P., Deep S. (2016). Effect of pH and temperature on conformational equilibria and aggregation behaviour of curcumin in aqueous binary mixtures of ethanol. RSC Adv..

[78] Martínez-Guerra J., Palomar-Pardavé M., Romero-Romo M., Corona-Avendaño S., Rojas-Hernández A., Ramírez-Silva M.T. (2019). New insights on the chemical stability of curcumin in aqueous media at different pH: Influence of the experimental conditions. Int. J. Electrochem. Sci..

[79] Stahn M., Ehlert S., Grimme S. (2023). Extended Conductor-like Polarizable Continuum Solvation Model (CPCM-X) for semiempirical methods. J. Phys. Chem. A..

[80] Madinah R., Rusydi F., Fadilla R.N., Khoirunisa V., Boli L.S.P., Saputro A.G., Hassan N.H., Ahmad A. (2023). First-principles study of the dispersion effects in the structures and keto–enol tautomerization of curcumin. ACS Omega.

[81] Vekeman J., Cuesta I.G., Faginas-Lago N., Wilson J., Sánchez-Marına J., Sánchez de Merás A. (2018). Potential models for the simulation of methane adsorption on graphene: Development and CCSD(T) benchmarks. Phys. Chem. Chem. Phys..

[82] Zou Y., Hou X., Wei H., Shao J., Jiang Q., Ren L., Wu J. (2023). Circumcoronenes. Angew. Chem. Int. Ed..

[83] Kelly C.P., Cramer C.J., Truhlar D.G. (2006). Aqueous solvation free energies of ions and ion-water clusters based on an accurate value for the absolute aqueous solvation free energy of the proton. J. Phys. Chem. B.

[84] Marenich A.V., Majumdar A., Lenz M., Cramer C.J., Truhlar D.G. (2012). Construction of Pourbaix diagrams for Ruthenium-based water-oxidation catalysts by density functional theory. Angew. Chem. Int. Ed..

[85] Solis B.H., Hammes-Schiffer S. (2014). Proton-coupled electron transfer in molecular electrocatalysis: Theoretical methods and design principles. Inorg. Chem..

[86] Isse A.A., Gennaro A. (2010). Absolute potential of the Standard Hydrogen Electrode and the problem of interconversion of potentials in different solvents. J. Phys. Chem. B.

[87] Yan L., Lub Y., Li X. (2016). A density functional theory protocol for the calculation of redox potentials of copper complexes. Phys. Chem. Chem. Phys..

