# Supplementary material for: Curcumin Electroanalysis at a Disposable Graphite Electrode
Source: Biosensors (Basel). 2025 Feb 23;15(3):137. doi: 10.3390/bios15030137 (PMC11940204; doi:10.3390/bios15030137)
Supplement: Supplementary file 1 [file biosensors-15-00137-s001.zip › biosensors-3461217-supplementary.pdf]

Curcumin Electroanalysis at a Disposable Graphite Electrode

Mihaela-Carmen Cheregi[1], Alina Tirsoaga[1], Cosmina Ion[1], Emilia-Elena Iorgulescu[1,*], Iulia Gabriela David[1,*] and Hassan Noor[2,3]

[1] Department of Analytical Chemistry and Physical Chemistry, Faculty of Chemistry, University of Bucharest, Panduri Av. 90-92, District 5, 050663, Bucharest, Romania; mihaela.cheregi@g.unibuc.ro (M.C.C.); alina.jurca@unibuc.ro (A.J.); cosmy.0408@yahoo.com (C.I.); emilia-elena.iorgulescu@chimie.unibuc.ro (E.E.I.): gabrielaiulia.david@g.unibuc.ro (I.G.D.)

[2] Department of Surgery, Faculty of Medicine, "Lucian Blaga" University Sibiu, Lucian Blaga Street 25,550169 Sibiu, Romania; nmfhassan@gmail.com

[3] Medlife-Polisano Hospital, Strada Izvorului 1A, 550172 Sibiu, Romania

* Correspondence: emilia-elena.iorgulescu@chimie.unibuc.ro (E.E.I.): gabrielaiulia.david@g.unibuc.ro (I.G.D.)

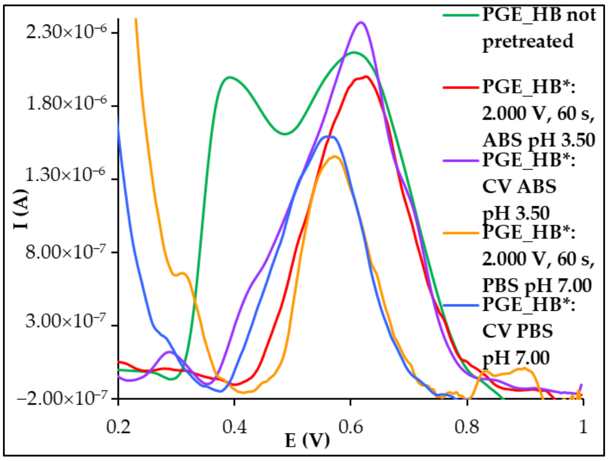

Figure S1. Differential pulse voltammograms recorded for 4.00×10^{-6} mol × L^{-1} CU in ABS pH 3.50, at PGE not activated (PGE_HB) and electrochemically pretreated (PGE_HB*) in different conditions, respectively.

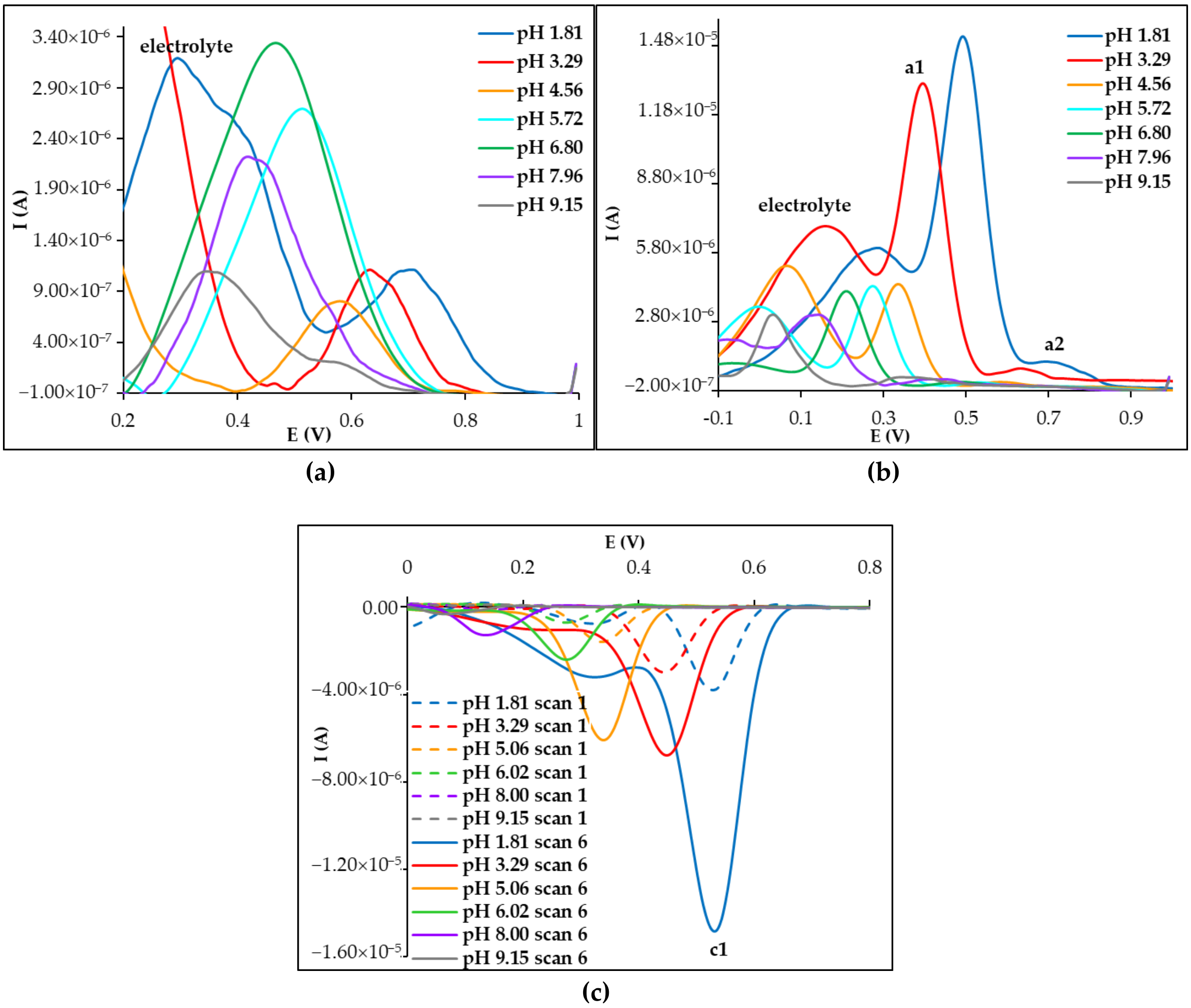

Figure S2. Differential pulse voltammograms recorded at PGE_HB* for 5.00 × 10⁻⁶ mol × L⁻¹ CU in BRB solutions with different pH values in: (**a**) anodic direction – scan 1; (**b**) anodic direction – scan 6 and (c) cathodic direction– scan 1 and scan 6.

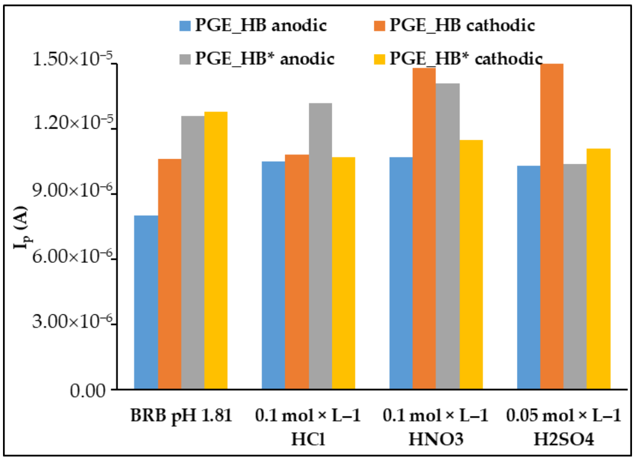

Figure S3. DPV peak currents recorded at the 6th scan for 5.00 × 10⁻⁶ mol × L⁻¹ CU in different acidic supporting electrolytes, at PGE_HB and PGE_HB*, respectively.

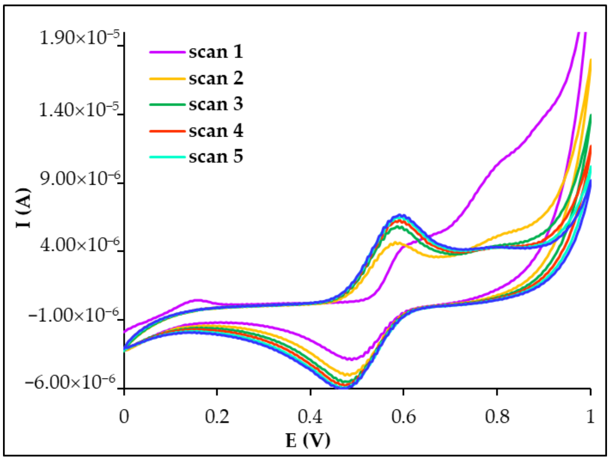

Figure S4. Repetitive cyclic voltammograms recorded at PGE_HB for 3.00×10^{-6} mol × L^{-1} CU in 0.05 mol × L^{-1} H_2SO_4, scan rate 0.100 V × s^{-1}.

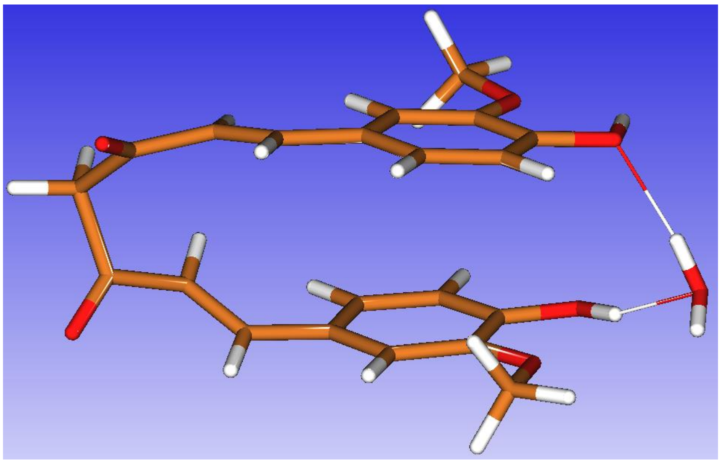

Figure S5. Stabilization of CU conformer 1 geometry by explicit solvation. The water molecule attaches through intramolecular hydrogen bonds to the phenolic -OH groups.

Table S1. The $E_p = f(pH)$ dependencies for CU voltammetric peaks recorded at disposable PGE.

scan		PGE_HB	PGE_HB*
anodic	1	–	E_{pa} (V) = –0.0485 × pH + 0.8011 R_2 = 0.9989
	6	E_{pa1} (V) = –0.0552 × pH + 0.5858 R_2 = 0.9946	E_{pa1} (V) = –0.0561 × pH + 0.5928 R_2 = 0.9981
	6	E_{pa2} (V) = –0.0467 × pH + 0.7977 R_2 = 0.9884	–
cathodic	1	E_{pc1} (V) = –0.0611 × pH + 0.6384 R_2 = 0.9985	E_{pc1} (V) = –0.0646 × pH + 0.6585 R_2 = 0.9963
	6	E_{pc1} (V) = –0.0608 × pH + 0.6351 R_2 = 0.9992	E_{pc1} (V) = –0.0640 × pH + 0.6582 R_2 = 0.9956

Table S2. Most stable conformers of CU β-diketone form in water solution: conformer geometry, relative energy difference and percentage from the overall conformers population (both calculated at xTB CPCM-X level), superposition between the geometries optimized at xTB CPCM-X (red) and B3LYP - def2-TZVP CPCM (cyan) levels; relative Gibbs energy difference (from B3LYP - def2-TZVP CPCM Hessian calculations).

Nr.	Conformer geometry found	ΔE (kcal/mol)	Population %	Superposition xTB vs. DFT	ΔG^0 (kcal/mol)
1	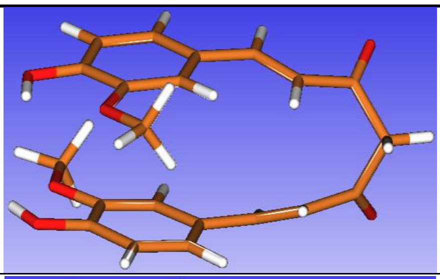	0	24.1	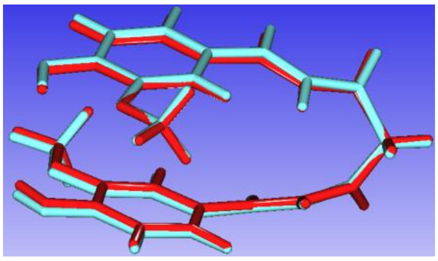	0
2	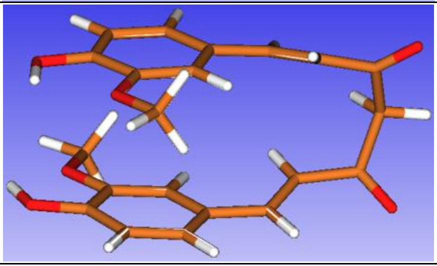	+0.37	13.0	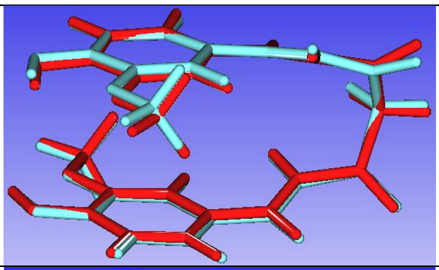	+0.003
3	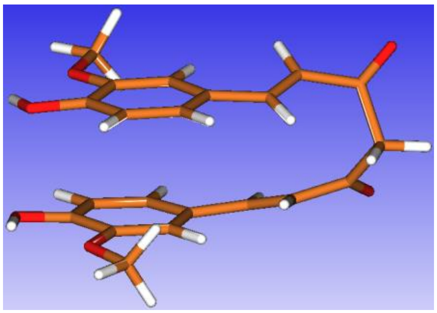	+0.48	10.8	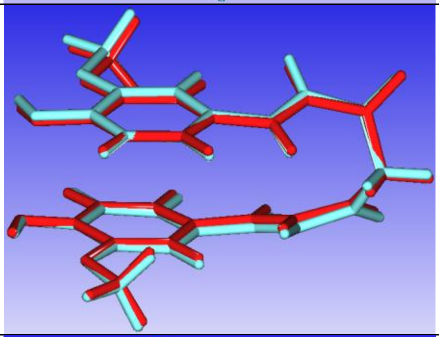	+0.03
4	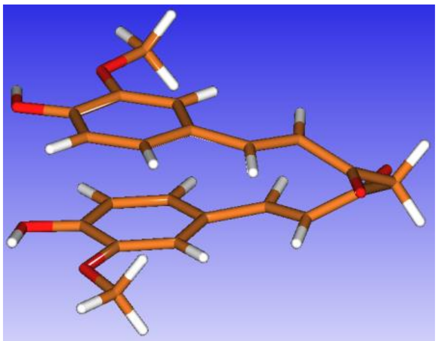	+0.56	9.3	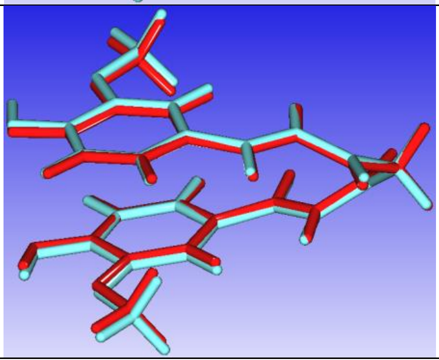	-0.3
